# Dose Optimization of Combined Linezolid and Fosfomycin against *Enterococcus* by Using an *In Vitro* Pharmacokinetic/Pharmacodynamic Model

**DOI:** 10.1128/Spectrum.00871-21

**Published:** 2021-12-01

**Authors:** Jun Mao, Ting Li, Na Zhang, Shuaishuai Wang, Yaowen Li, Yu Peng, Huiping Liu, Guang Yang, Yisong Yan, Lifang Jiang, Yanyan Liu, Jiabin Li, Xiaohui Huang

**Affiliations:** a Department of Basic and Clinical Pharmacology, School of Pharmacy, Anhui Medical University, Hefei, China; b Anhui Province Key Laboratory of Major Autoimmune Diseases, School of Pharmacy, Anhui Institute of Innovative Drugs, Anhui Medical University, Hefei, China; c Center for Drug Clinical Research, Shanghai University of Traditional Chinese Medicine, Shanghai, China; d Department of Infectious Diseases, The First Affiliated Hospital of Anhui Medical University, Hefei, China; INTHERES

**Keywords:** linezolid, fosfomycin, PK/PD model, *Enterococcus*, combination therapy

## Abstract

The rapid spread of antibiotic resistance among *Enterococcus* has prompted considerable interest in determining the dosage regimen of linezolid combined with fosfomycin. A checkerboard assay was employed to evaluate whether linezolid combined with fosfomycin had a synergistic effect on *Enterococcus* isolates from the hospital, including three drug-resistant strains (MIC of linezolid [MIC_LZD_], ≥8 mg/L; MIC of fosfomycin [MIC_FOF_], ≥256 mg/L). The *in vitro* static time-kill assay, dynamic pharmacokinetic (PK)/pharmacodynamic (PD) model, and semimechanistic PK/PD model were used to explore and predict effective combined dosage regimens. The checkerboard assay and *in vitro* static time-kill assay demonstrated that linezolid combined with fosfomycin has a synergistic effect on drug-resistant and sensitive *Enterococcus*. In the *in vitro* PK/PD model, the dosage regimen of linezolid (8 mg/L or 12 mg/L, steady-state concentration) combined with fosfomycin (6 g or 8 g) via a 0.5-h infusion every 8 h effectively suppressed bacterial growth at 24 h with a 3 log_10_ CFU/mL decrease compared with the initial inocula against two resistant and one sensitive *Enterococcus* isolates. The semimechanistic PK/PD model predicted that linezolid (more than 16 mg/L) combined with fosfomycin (6 g or 10 g) via a 0.5-h infusion every 8 h was required to achieve a 4 log_10_ CFU/mL decrease at 24 h against *Enterococcus* isolates (MIC_LZD_ ≥ 8 mg/L and MIC_FOF_ ≥ 256 mg/L). According to the prediction of the semimechanical PK/PD model, the effect of the combination was driven by linezolid, with fosfomycin enhancing the effect. Our study is the first to explore the synergistic effects of these two drugs from a qualitative and quantitative perspective and provides a simulation tool for future studies.

**IMPORTANCE** In this study, we found that linezolid combined with fosfomycin could kill *Enterococcus in vitro* and that the administered dose was significantly lower after the combination treatment, which could reduce adverse effects and the development of drug resistance. The potential mechanism of the two-drug combination against *Enterococcus* was revealed from a quantitative perspective, which is an important step toward dose optimization in simulated humans. We hope that our research will help build a better relationship between clinicians and patients as we work together to address the challenges of antibiotic resistance in the 21st century.

## INTRODUCTION

*Enterococcus* is one of the most common conditionally pathogenic Gram-positive bacteria. It can survive in harsh environments with antibiotics, which is a cause of hospital-acquired infections, causing urinary tract infections (UTIs), abdominal infections, endocarditis, and other infections associated with implanted medical devices (1, 2). Furthermore, the aminoglycosides recommended for the treatment of enterococcal infections often promote the development of bacterial resistance (3). Current challenges are likely to be resolved by the discovery of new antibiotics and other therapeutic approaches.

Linezolid (LZD) is used as a last-line drug for the treatment of severe enterococcal infections. However, long-term use of linezolid can result in thrombocytopenia, which limits dosage options (4). Additionally, acquired linezolid resistance genes have been increasingly reported in different enterococcal species and across different settings (5, 6). Hence, combination therapy has been proposed to alleviate the development of drug resistance and increase efficacy. Previous studies have confirmed that linezolid combined with fosfomycin (FOF) can effectively inhibit vancomycin-resistant and -sensitive *Enterococcus* strains (7, 8). However, these studies have been limited to the qualitative determination of the synergistic effects of the two drugs without a quantitative perspective on the mechanisms by which the drugs act on bacteria. Moreover, although the checkerboard and static time-kill assays are the most common methods used to detect synergistic effects of drugs because *in vitro* drug concentrations are static but *in vivo* drug concentrations are dynamic over time, it is difficult to guide clinical administration.

The establishment and development of *in vitro* dynamic pharmacokinetic/pharmacodynamic (PK/PD) models could help us address these questions. Boak et al. (9) investigated the administration of 600 mg linezolid every 12 h against vancomycin-resistant enterococci (VRE), which could significantly reduce bacterial infections at 24 h using the dynamic PK/PD model. However, in a recent Monte Carlo simulation, the effective killing of *Enterococcus* was observed only when 600 mg linezolid was administered every 8 h (10). In an *in vitro* bladder infection model, Abbott et al. (11) found that *Enterococcus* was significantly killed only when exposed to high peak concentrations of fosfomycin (maximum concentration of free, unbound drug in serum [*fC*_max_] > 1,000 mg/L). Such high drug concentrations could cause various side effects and the development of drug resistance, prompting us to seek new combined dosing regimens in an *in vitro* PK/PD model. To date, there have been no reports of the combined bactericidal activity of the two drugs in an *in vitro* dynamic model. Moreover, semimechanistic PK/PD modeling is a valuable tool that can be used to quantify concentration-effect curves and provide additional guidance for dose optimization (12). Computational models have been successfully established based on static bactericidal data for linezolid and dynamic bactericidal data for fosfomycin in an *in vitro* hollow-fiber infection model (13–15). However, these were all single-drug models, and no semimechanistic PK/PD models for coadministration have been developed nor have they predicted new dosing regimens. Additionally, most previous semimechanical PK/PD models based on bactericidal data from combined administration have focused on treating Acinetobacter baumannii, whereas there are few models for the combined treatment of *Enterococcus* (16–18).

In this study, *Enterococcus* strains that were sensitive and resistant to linezolid and fosfomycin were selected, and the checkerboard assay was used to detect whether linezolid combined with fosfomycin had a synergistic effect. On this basis, *in vitro* static and dynamic PK/PD time-kill experiments were designed to explore the combined dosing regimen of the two drugs. A semimechanical PK/PD model was developed to quantitatively explore the combined effects and dose optimization.

## RESULTS

### MICs and checkerboard and static time-kill assays.

The MICs of linezolid and fosfomycin against all *Enterococcus* isolates and the checkerboard assay results are shown in Table 1. Six strains were sensitive to linezolid (MIC range of 2 to 4 mg/L), and two strains were resistant to linezolid (MIC of linezolid [MIC_LZD_] = 8 mg/L). Only one strain was resistant to fosfomycin (MIC of fosfomycin [MIC_FOF_] = 256 mg/L), and the others were sensitive and intermediate bacteria (MIC range of 64 to 128 mg/L). Checkerboard results showed synergy (fractional inhibitory concentration index [FICI] ≤ 0.5) between linezolid and fosfomycin for seven isolates, with indifference (0.5 ≤ FICI≤ 4) observed for one isolate.

**TABLE 1:** MICs of antimicrobial agents against eight strains of *Enterococcus*

Strain	MIC (mg/liter)		MIC in combination	
	LZD	FOF	LZD+FOF	FICI
No. 1	4	64	1 + 16	0.50
No. 2	8	128	2 + 32	0.50
No. 3	2	128	1 + 16	0.625
No. 4	4	128	1 + 32	0.50
No. 6	2	256	0.5 + 64	0.50
No. 7	8	128	1 + 16	0.25
No. 8	2	128	0.5 + 32	0.50
No. 9	4	128	0.5 + 16	0.25
ATCC 29212	2	128	1 + 32	0.75

**FIG 1:**
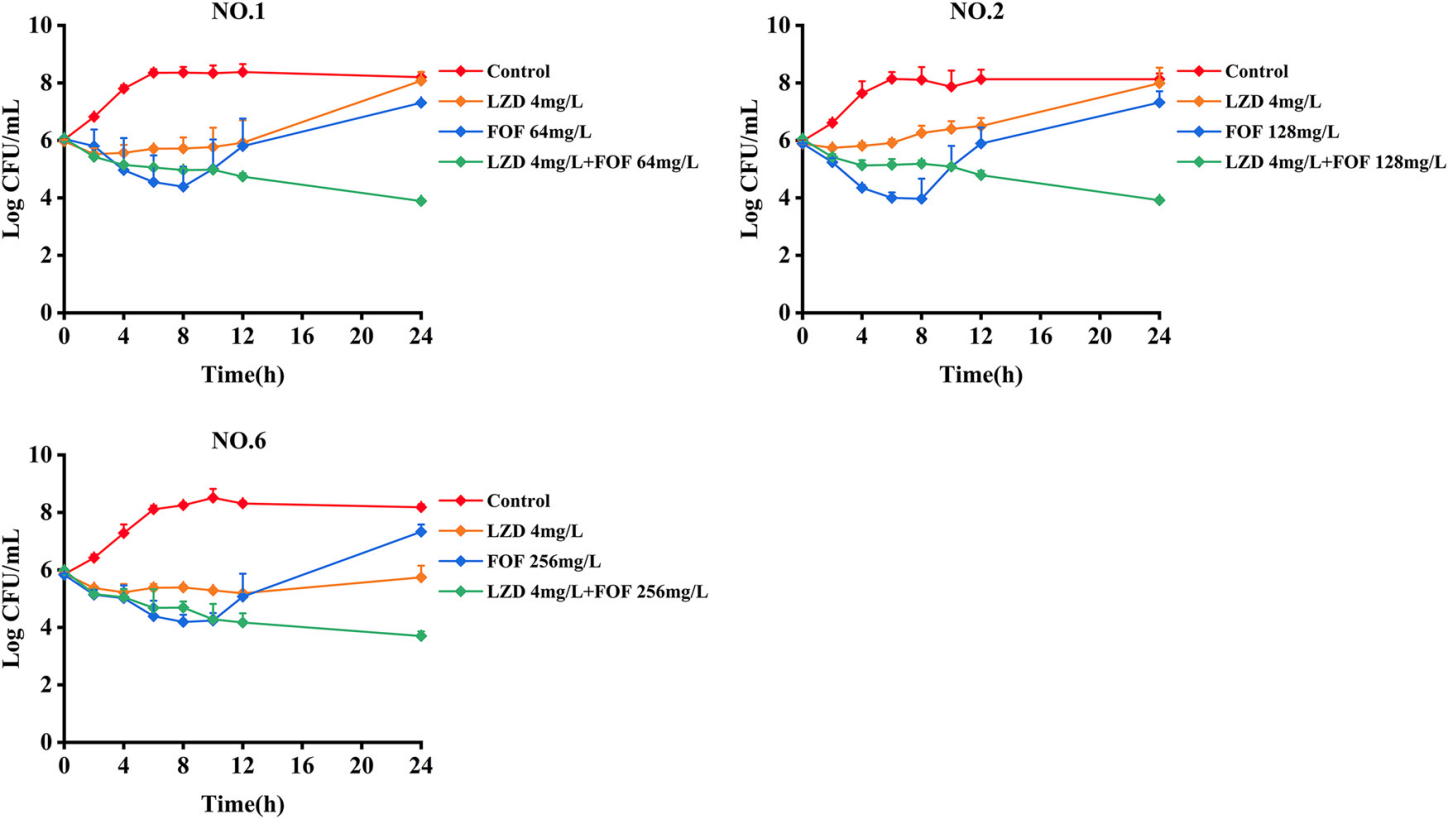
Static time-kill curves show the bactericidal effect of linezolid (orange), fosfomycin (blue), and their combination (green) against *Enterococcus* isolates (mean ± standard deviation [SD], *n =* 3). LZD, linezolid; FOF, fosfomycin.

The results of the static time-kill assay are shown in Fig. 1 and Table 2. Linezolid monotherapy (4 mg/L) produced little or no bacterial killing at any time, with growth close to control values at 24 h, except for in strain no. 6. With fosfomycin monotherapy at all concentrations (64, 128, or 256 mg/L), a bacterial killing to 2 log_10_ CFU/mL was observed across the first 4 to 8 h for all isolates. For all isolates, regrowth to control levels at 24 h subsequently occurred at all fosfomycin concentrations. Initial killing (0 to 8 h) with the linezolid and fosfomycin combinations resembled fosfomycin monotherapy. However, regrowth was suppressed in all isolates, with bacterial numbers never exceeding 4 log_10_ CFU across 24 h. After the combination, the changes in the number of bacteria after 24 h of treatment compared to the initial colony counts (ΔlogCFU_0–24_) values for strains no. 1, no. 2, and no. 6 were −2.18 ± 0.08, −2.13 ± 0.09, and −2.29 ± 0.13, respectively.

**TABLE 2:** ΔlogCFU_0–24_ values of fosfomycin and linezolid as monotherapy and in combination

Strain (MIC_FOF_/MIC_LZD_)	ΔlogCFU_0__–__24_ by antibiotic therapy (mean ± SD) (*n* = 3)			
	No drug	Fosfomycin	Linezolid	Combination
No. 1 (64/4)	2.17 ± 0.18	1.25 ± 0.12	2.11 ± 0.33	−2.18 ± 0.08
No. 2 (128/8)	2.15 ± 0.25	1.43 ± 0.38	2.11 ± 0.55	−2.13 ± 0.09
No. 6 (256/2)	2.34 ± 0.14	1.50 ± 0.26	−0.13 ± 0.43	−2.29 ± 0.13

### *In vitro* dynamic PK/PD model.

**FIG 2:**
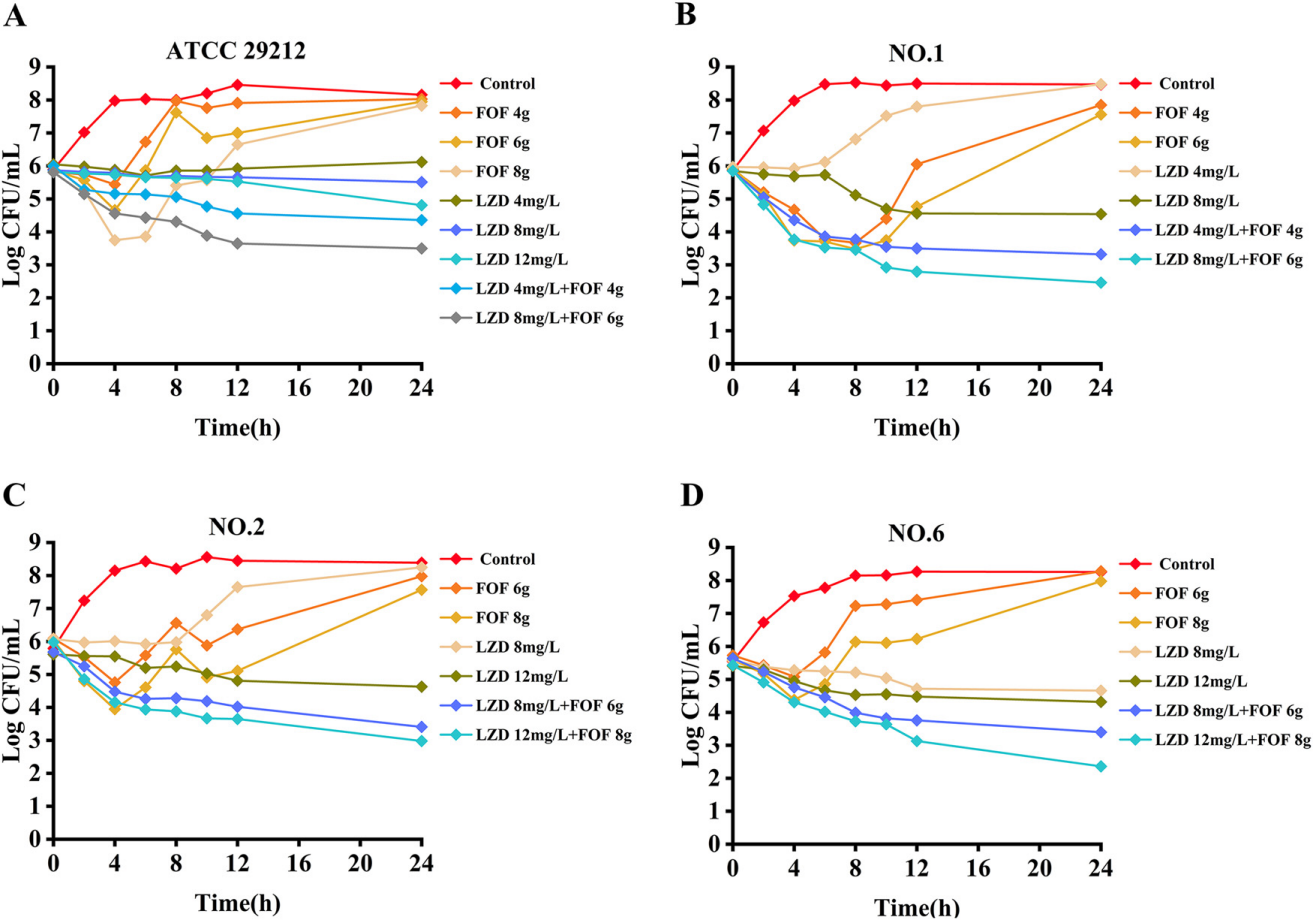
Dynamic *in vitro* PK/PD killing kinetics of strains ATCC 29212 (A), no. 1 (B), no. 2 (C), and no. 6 (D) in different dosage regimens. Fosfomycin doses of 4, 6, and 8 g were infused for 0.5 h every 8 h.

**FIG 3:**
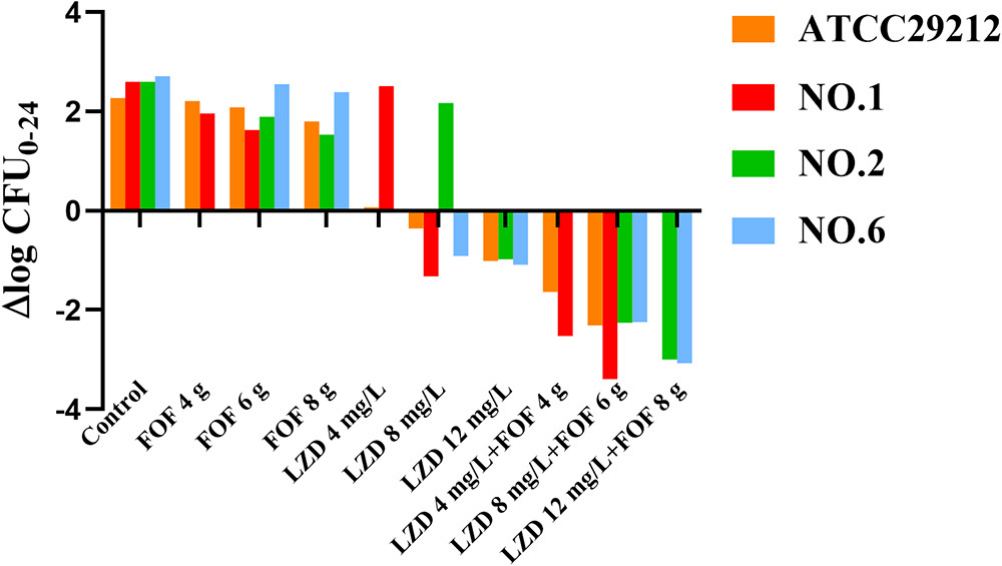
The values of ΔlogCFU_0-24_ for strains ATCC 29212, no. 1, no. 2, and no. 6 after each monotherapy and the combination.

The observed fosfomycin concentrations in the PK/PD model closely mimicked the targeted concentrations for the different simulated dosing regimens (see Fig. S3 in the supplemental material). The *in vitro* dynamic time-kill curves of linezolid and fosfomycin alone and in combination are shown in Fig. 2, and ΔlogCFU_0–24_ is shown in Fig. 3. Linezolid monotherapy (constant concentration of 12 mg/L) only had a bacteriostatic effect, with less than a 2 log_10_ CFU/mL reduction at 24 h. For linezolid-resistant *Enterococcus* strain no. 2 (MIC_LZD_ = 8 mg/L), linezolid at less than 8 mg/L failed to inhibit bacterial regeneration, and the colony count at 24 h was consistent with the growth group. Against the reference strain ATCC 29212 (MIC_FOF_ = 128 mg/L), fosfomycin monotherapy (8 g, a 0.5-h infusion every 8 h) produced an initial killing of >2 log_10_ CFU/mL at 4 h, followed by regrowth approaching that of the control at 24 h. Against the clinical strains (MIC_FOF_ = 128 mg/L or 256 mg/L), neither fosfomycin regimen (6 g with a 0.5-h infusion every 8 h or 8 g with a 0.5-h infusion every 8 h) produced initial killing at 4 h (maximum killing of <2 log_10_ CFU/mL) and regrowth to control values at 24 h. For the fosfomycin-sensitive *Enterococcus* strain no. 1 (MIC_FOF_ = 64 mg/L), fosfomycin monotherapy (6 g, a 0.5-h infusion every 8 h) displayed a maximum killing of >2 log_10_ CFU/mL at 4 h and persisted up to 8 h, with slow regrowth to 7 log_10_ CFU/mL thereafter.

In contrast, the combination of linezolid and fosfomycin demonstrated stronger activity than the two drugs alone, with bactericidal effects against ATCC 29212 and three clinical isolates. Combination therapy against these strains produced similar initial killing to fosfomycin monotherapies across the first 4 h, but from 4 h onwards, no regrowth bacteria were detected, and the colony count continued to drop. For two clinical isolates (no. 2 and no. 6), linezolid (12 mg/L) plus fosfomycin (8 g infused for 0.5 h every 8 h) resulted in a >3 log_10_ CFU/mL reduction at 24 h. For clinical isolate no. 1, linezolid (8 mg/L) combined with fosfomycin (6 g infused for 0.5 h every 8 h) had a strong bactericidal effect, and the lowest concentration of linezolid (4 mg/L) with fosfomycin (4 g infused for 0.5 h every 8 h) could provide a reduction of nearly 3 log_10_ CFU/mL at 24 h. All remaining combinations produced similar killing effects against all strains within 24 h, consistently inhibiting bacterial regeneration, with no growth beyond 4 log_10_ CFU/mL at 24 h.

### Semimechanical PK/PD model.

**FIG 4:**
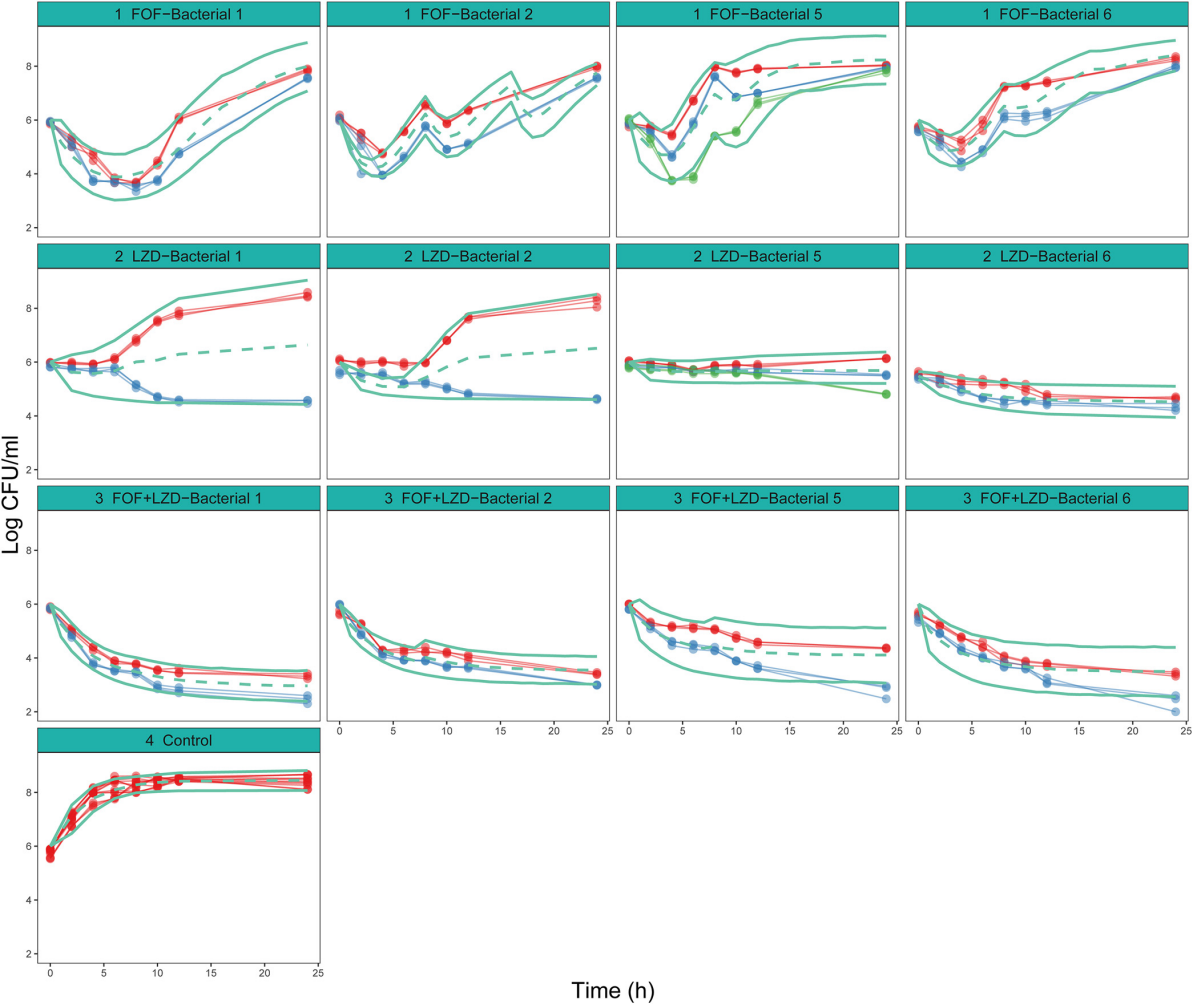
Visual predictive check of each PK/PD model. Solid points represent observed bacterial counts (the values of the same batch were tested three times). Points linked by a line are from the same arm. Different colors represent different dosage regimens. Green lines are the model-predicted 5th, 50th, and 95th percentiles of bacterial counts. From left to right, the four columns of the figure represent bacterial strains no. 1, no. 2, ATCC 29212, and no. 6, respectively. FOF+LZD, fosfomycin combined with linezolid; Control, no drug.

**FIG 5:**
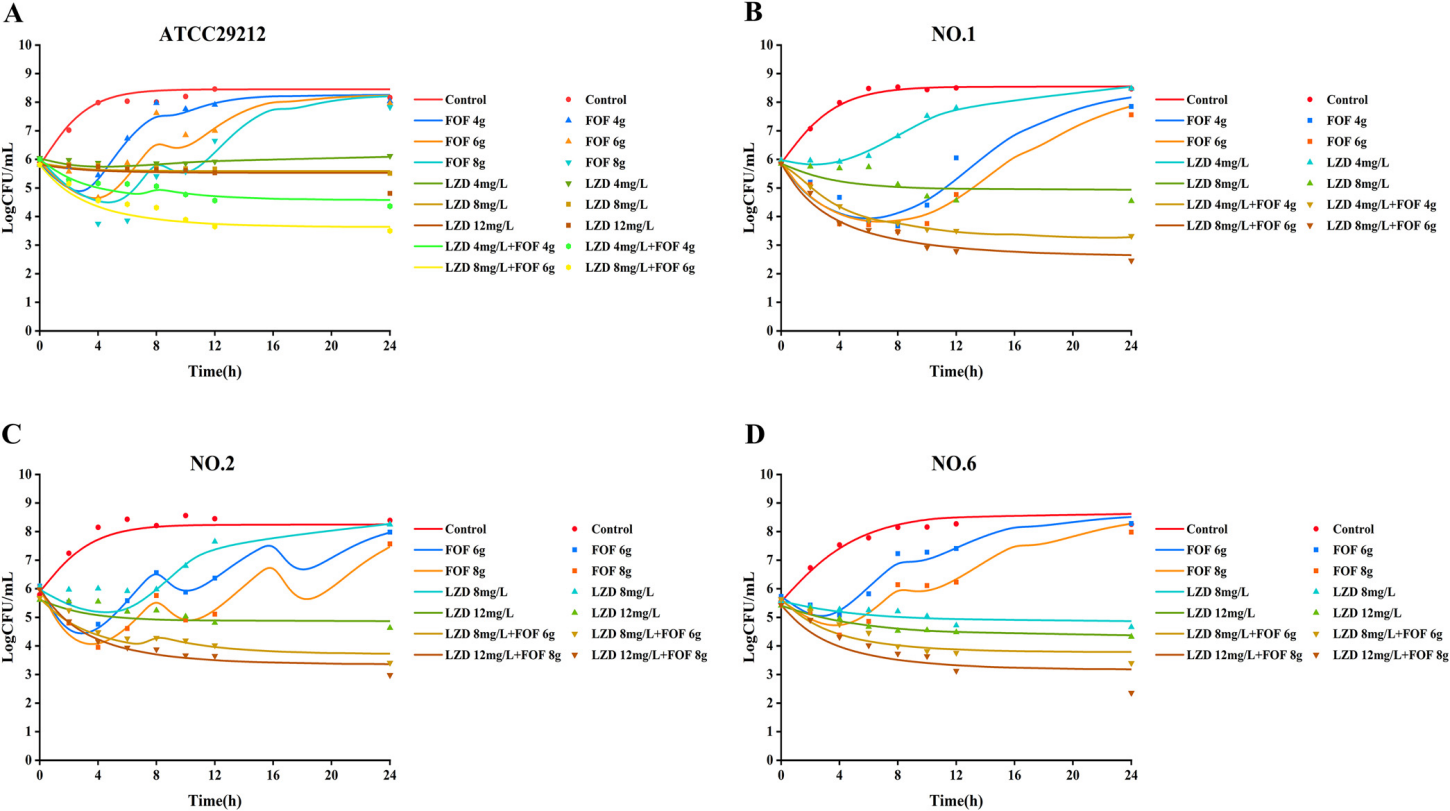
Observed (symbols) and model fitted (lines) viable counts for the dynamic *in vitro* PK/PD model experiments with fosfomycin or linezolid alone and the combination against *Enterococcus* strains ATCC 29212 (A), no. 1 (B), no. 2 (C), and no. 6 (D). Fosfomycin doses of 4, 6, and 8 g were infused for 0.5 h every 8 h.

The time course of changes in bacterial counts from baseline was well described by the semimechanical PK/PD model. The goodness-of-fit plots indicated a relatively good fit with the observed data (see Fig. S4 in the supplemental material). Specifically, plots of observation (OBS) versus population prediction (PRED) and observation (OBS) versus individual prediction (IPRED) were symmetrically distributed and close to the identity line, implying good predictions. The plots of conditional weighted residual errors (CWRES) versus PRED and plots of CWRES versus time showed no trend and were randomly scattered around the identity line at CWRES = 0, indicating the suitability of the error model for this study. The visual predictive check (VPC) plots indicated that the 95% confidence interval (CI) of the model prediction covered almost all of the observed data, demonstrating good predictability by the model (Fig. 4). A comparison between the predicted and observed values of the model is shown in Fig. 5, which shows that the observed values are distributed around the fitted prediction curve, and there are not particularly prominent and inconsistent values. The estimated parameters are listed in Table 3.

**TABLE 3:** Parameter estimates for the *in vitro* PK/PD model

Parameter	Explanation	Value for strain:			
		ATCC 29212	No. 1	No. 2	No. 6
*K_g_* (h^−1^)	Rate constant of bacterial net growth	0.474	0.452	0.557	0.315
*B*_max_ (log_10_ CFU/mL)	Bacterial count in the stationary phase	8.25	8.55	8.45	8.63
*E*_max-LZD_ (h^−1^)	Maximum achievable kill rate constant by linezolid	0.158	0.191	0.236	0.174
EC_50-LZD_ (mg/L)	Linezolid concn that results in 50% of *E*_max_	0.114	3.95	4.81	1.71
*E*_max-FOF_ (h^−1^)	Maximum achievable kill rate constant by fosfomycin	0.256	0.294	0.415	0.192
EC_50-FOF_ (mg/L)	Fosfomycin concn that results in 50% of *E*_max_	19.7	0.0135	23.5	28.1
Hill_LZD_	Hill factor for linezolid	2.98	187	50	2.06
Hill_FOF_	Hill factor for fosfomycin	7.53	3.63	2.34	3.7
*f*_LZD_	Maximal adaptation factor for linezolid	21.9	5.29	0.765	1.45
*k*_LZD_	Rate of adaptation for linezolid	0.0547	0.0001	0.0312	1.41
*f*_FOF_	Maximal adaptation factor for fosfomycin	54.2	68,600	7.53	38.1
*k*_FOF_	Rate of adaptation for fosfomycin	0.0001	0.0001	0.000712	0.0001
Int	Parameter describing drug interaction	1.29	−1.96	1.37	2.08

Among all strains, the linezolid-resistant strain no. 2 had the fastest growth rate (*K_g_* = 0.557 h^−1^), whereas the fosfomycin-resistant strain no. 6 had the slowest growth rate (*K_g_* = 0.315 h^−1^), and the other two strains had a *K_g_* of 0.474 h^−1^ and 0.452 h^−1^. Among the two resistant strains, linezolid and fosfomycin had the highest 50% effective concentration (EC_50_) (4.81 mg/L and 28.1 mg/L, respectively) relative to other sensitive bacteria. Compared with susceptible bacteria and ATCC 29212, linezolid had the highest maximum effect (*E*_max_) (0.236 h^−1^) in the linezolid-resistant strain, whereas fosfomycin had the lowest *E*_max_ (0.192 h^−1^) in the fosfomycin-resistant strain. Strains that were more sensitive to linezolid and fosfomycin, such as ATCC 29212 and no. 1, showed the highest maximum adaptive resistance factor (*f*_LZD_ = 21.9 and *f*_FOF_ = 68,600).

**FIG 6:**
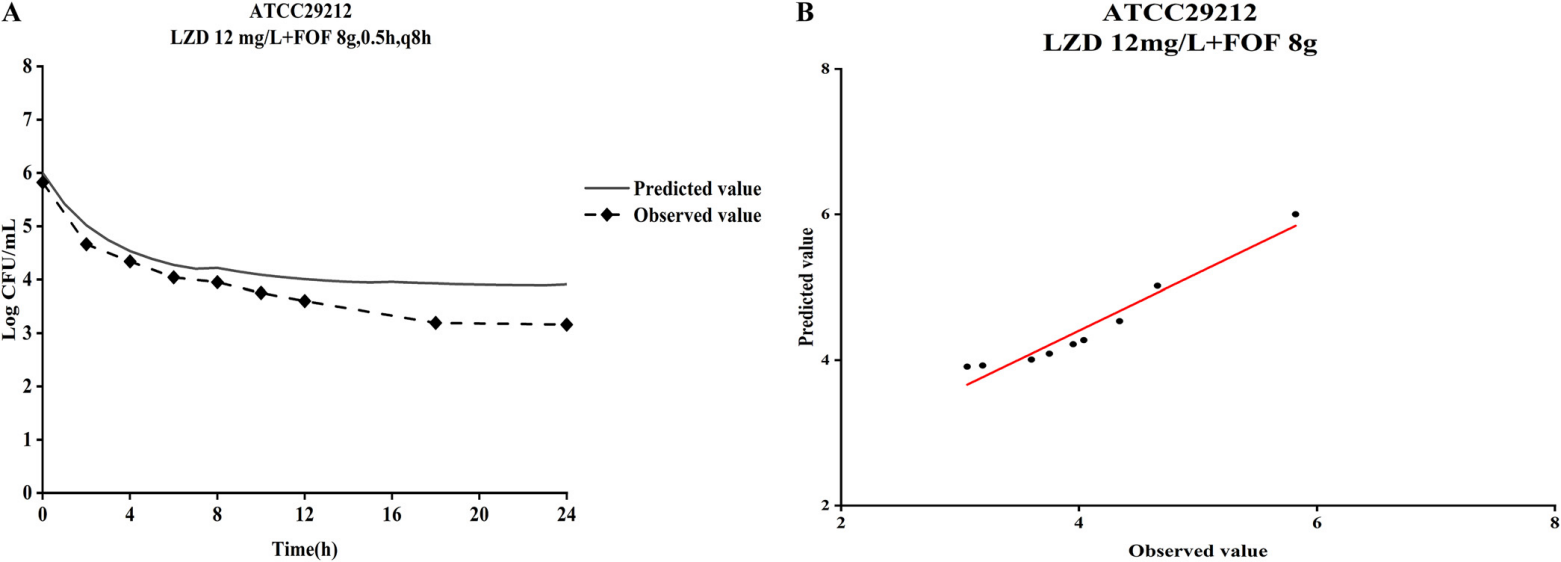
Validation of the PK/PD modeling for the regimen of 12 mg/L linezolid in combination with 8 g fosfomycin every 8 h with a 0.5 h infusion.

**FIG 7:**
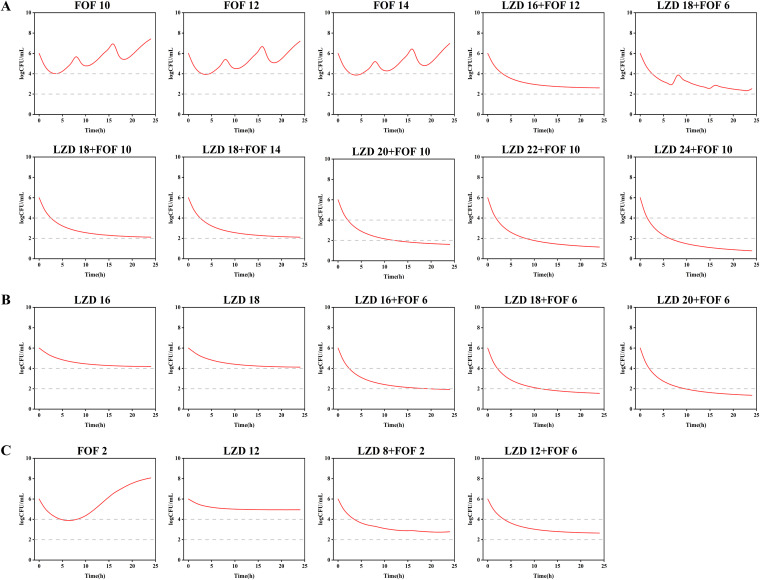
Pharmacodynamic predictions of linezolid and fosfomycin mono and combination therapy against *Enterococcus* strains no. 2 (A), no. 6 (B), and no. 1 (C). The units for linezolid and fosfomycin are milligrams per liter at steady state and grams with a 0.5-h infusion, respectively.

Linezolid (12 mg/L) combined with fosfomycin (8 g with a 0.5-h infusion every 8 h) against ATCC 29212 was used as an external validation scheme, using estimated parameters from ATCC 29212 to simulate the pharmacodynamics of this dose combination and compared with observed values. As shown in Fig. 6, predicted and observed values had a consistent downward trend, but the predicted values underestimated the bactericidal effect of the combined dosing regimen, and the difference with the observed values ranged from 1 log_10_ CFU/mL. Based on the successfully validated model, we predicted new therapies for the three clinical isolates (Fig. 7). The dosage of fosfomycin increased from 8 g to 14 g for all strains, but there was no significant increase in bactericidal effect, and the trend remained essentially the same. Even if the linezolid concentration alone was increased to 24 mg/L, it only provided continuous bacterial inhibition and had no bactericidal effect. However, a good synergistic bactericidal effect in the combined regimens was observed. For strain no. 2, linezolid (18 mg/L) combined with fosfomycin (10 g with a 0.5-h infusion every 8 h) had a 4 log_10_ CFU/mL reduction at 24 h relative to the initial inoculum. When the linezolid concentration was increased to 24 mg/L, coadministration provided a greater than 5 log_10_ CFU/mL reduction at 24 h. For strain no. 6, linezolid (more than 16 mg/L) combined with fosfomycin (6 g with a 0.5-h infusion every 8 h) at 24 h reached a >4 log_10_ CFU/mL decrease. Additionally, for strain no. 1, linezolid (8 mg/L) plus the lowest dose of fosfomycin (2 g) displayed a 3 log_10_ CFU/mL reduction at 24 h, whereas there was no significant change in the bactericidal effect after increasing the dose.

## DISCUSSION

In this study, static and dynamic time-kill experiments *in vitro* confirmed that linezolid combined with fosfomycin killed susceptible *Enterococcus* strains and *Enterococcus* strains resistant to linezolid or fosfomycin, and potential dosage regimens were discussed. The semimechanical PK/PD model predicted the killing of *Enterococcus* strains under multiple dosing schedules and provided a new method for optimizing combination therapies.

The results of *in vitro* dynamic time-kill curves and the parameters of the semimechanism PK/PD model intuitively showed that linezolid combined with fosfomycin had a synergistic bactericidal effect on *Enterococcus*. This synergy likely occurs because fosfomycin can interfere with the first step of mucopeptide synthesis in the bacterial cell wall, helping linezolid enter the bacteria and produce antibacterial effects (19). Linezolid, a unique synthetic antimicrobial of oxazolidinone, blocks the formation of the initiation complex at the beginning of the translation process by binding to the 50S large subunit of 23S rRNA (20). According to the *in vitro* dynamic time-kill curve, the maximum bactericidal rate of coadministration was not significantly increased relative to fosfomycin at 4 to 6 h, but the bactericidal effect became progressively stronger after 6 h. This might occur because linezolid limits the rapid bactericidal effect of fosfomycin during early bacterial reproduction, but in later stages, when bacterial populations reach a stable period, the combination of the two drugs produces a more potent bactericidal effect. Moreover, in a previous study, we found that the combination of linezolid and fosfomycin against *Enterococcus* could effectively close the resistance mutation-selection windows of the other, which is a potential mechanism to explain the ability of coadministration to inhibit bacterial regeneration at a later stage (8). Additionally, the production of enterococcal biofilms in an *in vitro* model protected a subpopulation of bacterial inoculum from fosfomycin, thereby regenerating a subpopulation when fosfomycin concentration was below the MIC of the isolates (11). It has been reported that linezolid and fosfomycin individually inhibit the growth of enterococcal biofilms, perhaps with a stronger inhibition after the combination (21, 22). However, further study of the synergistic mechanism of linezolid and fosfomycin from metabolomics is necessary. Additionally, there is no pharmacokinetic interaction between linezolid and fosfomycin (23, 24). Linezolid and fosfomycin are both excreted from the urine in parent form through glomerular filtration and are almost eliminated in the renal pathway (25). Thus, linezolid combined with fosfomycin has little effect on the pharmacokinetics of the other, but further animal and human studies are warranted.

Combination dosing regimens remain a trusted therapy for drug-resistant bacteria. As mentioned earlier, when linezolid has to be used, infection from linezolid-resistant *Enterococcus* is unavoidable (26). These resistance mutations include vertical transmission mutations at linezolid targets; 23S rRNA gene sequences; alterations in the ribosomal proteins L3, L4, and L22; and the efflux pump genes *optrA* and *poxtA* (27). Fosfomycin generally has a very high MIC for *Enterococcus* and is recommended for oral use in treating UTIs, whereas intravenous and combination therapies are usually used for severe infections (28). The presence of the *fosB* gene, the mutation of the fosfomycin target enzyme MurA, and the high-level expression of *fosX* make *Enterococcus* resistant to fosfomycin (29). It is important to note whether the combination has synergies if the strain is resistant to at least one drug. Therefore, linezolid-resistant Enterococcus faecium and fosfomycin-resistant E. faecium were included in the experiment to evaluate whether the combination of linezolid and fosfomycin could have a bactericidal effect at low doses. For linezolid-resistant E. faecium strain no. 2 and fosfomycin-resistant E. faecium strain no. 6, linezolid (8 mg/L) plus fosfomycin (6 g with a 0.5-h infusion every 8 h) could achieve >2 log_10_ CFU/mL killing at 24 h. For fosfomycin-resistant E. faecium strain no. 6, killing effects approaching a 4 log_10_ CFU/mL decrease could be achieved when the combined administration dose increases. Therefore, in fosfomycin-resistant *Enterococcus*, combined linezolid administration is recommended to obtain a better bactericidal effect. Studies have shown that when the drug concentration is close to or higher than the MIC, it is easy to promote bacterial drug resistance (9). However, this combination option is good for patients who may not have access to effective drugs.

The semimechanical PK/PD model parameters provide quantitative data to understand further how drugs act on bacteria and bacterial resistance. For linezolid-resistant E. faecium strain no. 2, linezolid and fosfomycin had the highest *E*_max_ relative to the other three strains, probably because the *K_g_* of this strain was the fastest. Both linezolid and fosfomycin had the highest EC_50_ values for their respective resistant strains relative to the other sensitive strains. This could indicate that in low-degree resistant *Enterococcus*, higher drug concentrations are often required to achieve maximum bactericidal rates, and it is difficult to achieve good bactericidal efficacy at low concentrations with monotherapy. The maximum bactericidal rate *E*_max_ from the estimated parameters is positively correlated with the bacterial growth rate *K_g_*. This could explain why linezolid combined with fosfomycin did not have a good initial bactericidal rate in the early stage because linezolid inhibited the bacterial growth rate, which also inhibited the *E*_max_ of fosfomycin. However, the combination may have lowered the EC_50_ of the other, which explains why a combination at low concentrations could have a good bactericidal effect. In terms of bacterial resistance, the maximum adaptive resistance factor *f* increases with a decrease in EC_50_, indicating that high exposure and continuous administration increase resistance if given before the bacteria return to their susceptible phase (30). Additionally, drug-sensitive strains usually have higher *f* values, which raises a warning for monotherapy, because this could easily promote the development of bacterial resistance. According to the parameters predicted by the semimechanical PK/PD model, three strains had an interaction (int) of >0, except for strain no. 1. This implies that the model considers coadministration to be nonsynergistic for the no. 1 strain of the four strains. However, according to the checkerboard assay and time-kill curve results, the combined administration had a synergistic effect on strain no. 1. These methods define synergism and antagonism in numerical changes by comparing the bacterial colonies at 24 h, whereas they ignore the effect of drugs and bacteria long-term, and synergism cannot be judged only by the change at a certain moment. It is fully realized that there are many ways to express synergism, which is related to the specificity of the strain and the detection method. Additionally, the clinical success of treatment depends on the overall activity of the infected site against the infected pathogen (31). The clinician considers the removal of pathogenic microorganisms and whether the patient can be cured. Therefore, the *in vitro* bactericidal effects should be evaluated by considering the differences *in vivo*.

The established semimechanical PK/PD model was used to predict new combination regimens of three clinical isolates of *Enterococcus.* Linezolid has been clinically proven to be safe and effective with a minimum concentration (*C*_min_) between 2 and 8 mg/L and a maximum concentration (*C*_max_) between 10 and 20 mg/L, and it can easily cause adverse reactions such as bone marrow suppression and thrombocytopenia if the concentration exceeds 20 mg/L (32). The recommended dose of linezolid of 600 mg every 12 h (q12h) achieves steady-state peak concentrations between 12 and 20 mg/L in a population with normal renal function (33). The average duration of treatment with linezolid for enterococcal infections is typically 26 d, and steady-state concentrations are typically achieved at 2 to 4 administered doses; thus, we discuss the bactericidal effects of steady-state concentrations under prolonged treatment (34). For linezolid-resistant E. faecium strain no. 2, because of the low *E*_max_ and high EC_50_ value, increasing the linezolid concentration alone has only a sustained bacteriostatic effect without a significant bactericidal effect, with colony values barely below 4 log_10_ CFU/mL at 24 h. In the rat bacteremia model, Abdelhady and Mishra administered 120 mg/kg of body weight to simulate a human blood concentration of 600 mg q12h and observed colony values of approximately 4 log_10_ CFU/mL in various tissues and organs after 4 d (35). In a rabbit endocarditis model, the administration of 10 mg/kg/12 h in simulated humans revealed only a 2 log_10_ CFU/mL decrease in colony count even after 4 d (36). In contrast, in rat peritonitis simulating standard dose administration, linezolid was bactericidal and bacteriostatic against vancomycin-resistant *Enterococcus*, and the bactericidal effect *in vivo* may be caused by the synergistic effects of linezolid with intrinsic host defenses (polymorphonuclear leukocytes [PMNs]) or molecules (e.g., host defense peptides and antibodies) *in vivo* (37). Because standard dosing in critically ill patients still shows failure, the issue of whether to change the dose of linezolid is still being discussed. Partial Monte Carlo simulations using the cumulative percentage of a 24 h period that the concentration is above MIC (%*T*) > MIC above 85% as a PK/PD target suggests that *Enterococcus* treatment should be a dose of 600 mg q8h or 600 mg q6h (10, 20, 38). Doses administered at 1,800 mg and 2,400 mg per day will inevitably increase the steady-state peak concentration and be out of the safe range. Thus, the model simulated the linezolid concentration at 20 mg/L and found that linezolid alone still showed inhibition and no significant enhancement of the bactericidal effect. This may be because of its low *E*_max_ value, which limits its ability for rapid bactericidal activity.

However, both sensitive and low-degree resistant bacteria will develop high resistance after prolonged administration. Therefore, an old antibiotic (fosfomycin) was added with linezolid for a stronger bactericidal effect. Because of the high MIC of fosfomycin in *Enterococcus* and the area under the concentration-time curve for the free, unbound fraction of a drug (*f*AUC)/MIC is considered to be the most relevant PK/PD indicator, fosfomycin is usually administered at doses of 12 to 24 g daily to maintain very high blood concentrations, but this could produce excess sodium and hypokalemia in patients (39, 40). These regimens were for strains with MICs below 32 mg/L, whereas the strains in our experiments were above 64 mg/L, which may require higher loading doses and daily maintenance doses. Fosfomycin alone continued to increase the dose up to 14 g, and the initial maximum bactericidal effect was not more than a 2 log_10_ CFU/mL reduction. This is consistent with the findings of the *in vitro* PK/PD model of Abbott et al., and it is difficult to attain a bactericidal role if the *fC*_max_ is above 1,000 mg/L (11). Although clinical data suggest that 12 to 24 g of fosfomycin daily is usually the treatment for serious infections caused by Gram-negative bacteria, coadministration is usually recommended for serious infections caused by *Enterococcus*. For linezolid-resistant E. faecium strain no. 2, combined administration of linezolid (18 mg/L) and fosfomycin (6 g) was predicted to have a bactericidal effect with more than a 3 log_10_ CFU/mL decrease at 24 h after prediction. For fosfomycin-resistant E. faecium strain no. 6, the bactericidal effect of increasing the linezolid concentration was much greater than that of increasing the dose of fosfomycin. Linezolid (16 mg/L) combined with fosfomycin (6 g) could attain below 2 log_10_ CFU/mL at 24 h. Because the model judged that the combination of the two drugs had an antagonistic effect on strain no. 1, a ΔlogCFU_0–24_ > 4 could not be achieved even if the dosage was increased. However, the model predicted that linezolid (8 mg/L) combined with fosfomycin (2 g) could achieve the greatest effect. In treating sensitive bacterial infections, a combination of drugs at low doses can have a significant bactericidal effect. A very good synergistic effect of the two drugs was observed in the predicted results of our model. Higher concentrations of linezolid (>20 mg/L) combined with fosfomycin did not significantly improve the bactericidal effect compared with linezolid (below 20 mg/L) combined with fosfomycin. We believe it is safe and effective to maintain the recommended dose of 600 mg q12h of linezolid in combination with a low dose of fosfomycin in a coadministration.

To our knowledge, this is the first semimechanistic PK/PD model to investigate linezolid-fosfomycin combinations against *Enterococcus*. From a quantitative point of view, the mechanism of linezolid and fosfomycin inhibiting *Enterococcus* was explained, and it was determined that linezolid played a major role in combination administration. Therefore, a high dose of linezolid should be administered in combination therapies to produce a better bactericidal effect. The external validation results of the model were good and can be used as a simulation tool in future studies. For drug-resistant bacteria, the model predicted that linezolid in the safe concentration window (10 to 20 mg/L), combined with fosfomycin at 6 g or 10 g every 8 h, could achieve a 4 log_10_ CFU/mL reduction at 24 h. However, the limitation of this study is that the linezolid simulation scheme is the steady-state concentration after administration of 600 mg q12h, which hinders the study of pharmacodynamics under real-time changes in concentration. Therefore, future studies should focus on selecting multiple strains, combining *in vivo* and *in vitro* experiments, simulating additional dosing regimens of linezolid, and developing complete semimechanistic PK/PD models to simulate administration in different populations.

## MATERIALS AND METHODS

### Bacterial strains, medium, and antibiotics.

Eight nonduplicate clinical isolates of *Enterococcus* were isolated from urine and blood at the First Affiliated Hospital of Anhui Medical University. These strains were not collected specifically for this study and were approved by the hospital. All strains were identified using the automated Vitek-2 system (bioMérieux, Marcy l’Etoile, France). ATCC 29212 was used as the quality control strain.

Linezolid and fosfomycin were purchased from the National Institute for Food and Drug Control of China (Beijing, China). Glucose-6-phosphate was purchased from Sigma-Aldrich. Mueller-Hinton broth (MHB) (Oxoid, England) and Mueller-Hinton Agar (MHA) (Oxoid, England) were used for susceptibility, checkerboard, and time-kill assays.

### Determination of antimicrobial susceptibility and checkerboard assay.

The MICs of tested antibiotics against *Enterococcus* were determined using the agar dilution method following the Clinical and Laboratory Standards Institute (CLSI) guidelines (41). Single colonies were selected from *Enterococcus* cultured overnight and diluted to ∼5 × 10^5^ CFU/mL by adding a 0.9% NaCl solution. Bacteria were inoculated into a medicated agar plate prepared by the 2-fold dilution method and then incubated at 37°C for 24 h. The fosfomycin agar plate also included glucose-6-phosphate (G-6-P) for a final concentration of 25 mg/L. The MIC was defined as the lowest drug concentration without visible colony growth. ATCC 29212 was used as the quality control strain for each batch, and the experiment was repeated three times.

The synergistic effects of linezolid and fosfomycin combinations at different concentrations were evaluated using a checkerboard assay. The linezolid concentration was 0.03125 mg/L to 8 mg/L, and the concentration of fosfomycin was 0.5 mg/L to 256 mg/L. Each strain was inoculated in 96-well plates to obtain a suitable bacterial suspension (∼5 × 10^5^ CFU/mL) at a final volume of 200 μL (25 mg/L G-6-P) and incubated at 37°C for 18 to 22 h. All experiments were performed in triplicate.

The fractional inhibitory concentration index (FICI) is defined as follows: FICI = (MIC of drug A in combination/MIC of drug A alone) + (MIC of drug B in combination/MIC of drug B alone). The interpretation of FICI against *Enterococcus* was as follows: FICI ≤ 0.5, synergy; 1 < FICI ≤ 4, indifference; FICI > 4, antagonism (19).

### Static time-kill assays.

Linezolid combined with fosfomycin for static time-kill assays for strains no. 1 (Enterococcus faecalis), no. 2 (linezolid-resistant Enterococcus faecium), and no. 6 (Fosfomycin-resistant Enterococcus faecium). The assay was performed according to previously published methods (42). In short, the initial inoculation amount of bacteria was ∼1 × 10^6^ CFU/mL in a 10 mL MHB system, the designed linezolid concentration was 4 mg/L, and the fosfomycin concentrations were 64, 128, and 256 mg/L. Sampling and counting were performed at 0, 2, 4, 6, 8, 10, 12, and 24 h. The assay was performed in triplicate. The synergistic effect was defined as a reduction of more than 2 log_10_ CFU/mL at 24 h compared with the most active single drug.

### *In vitro* PK/PD model.

The *in vitro* PK/PD model used in the dynamic time-kill experiment has been described previously (43). In the PK/PD studies, the reference strain ATCC 29212 was included in addition to the three isolates used in static time-kills. Linezolid was simulated at steady-state plasma concentrations in healthy adults after 7 d of continuous administration of 600 mg q12h, with a maximum concentration (*C*_max_) of 15.7 mg/L, a minimal concentration (*C*_min_) of 3.84 mg/L, an area under the concentration-time curve from 0 to 12 h (AUC_0–12_) of 93.4 mg h/L, and a half-life (*t*_1/2_) of 4.8 h. The plasma protein binding rate was 31% (44). According to the results of static time-kill experiments and the steady-state concentration range, the concentrations of simulated linezolid were 4, 8, and 12 mg/L. For fosfomycin monotherapy, Phoenix WinNonlin software was used to determine the two-compartment model (see Table S1 in the supplemental material) according to data on fosfomycin concentrations in human blood, and three dosages of 4 g, 6 g, and 8 g were simulated with the administration of a 0.5-h infusion every 8 h (45). The flow rate through the system was set to achieve the desired half-life at β phase (*t*_1/2β_) for each regimen; the simulated *t*_1/2β_ at 3.3 h, AUC_0–8_ of 383, 575, and 766 mg h/L, and *C*_max_ of 160.2, 240.3, and 320.4 mg/L mimicked those observed in healthy volunteers receiving the equivalent fosfomycin regimens. For combination therapy, linezolid (4 mg/L and 8 mg/L) was combined with fosfomycin (4 g administered with a 0.5-h infusion every 8 h or 6 g administered with a 0.5-h infusion every 8 h) against ATCC 29212 and no. 1 strains. Linezolid (8 mg/L and 12 mg/L) was combined with fosfomycin (6 g administered with a 0.5-h infusion every 8 h or 8 g administered with a 0.5-h infusion every 8 h) against no. 2 and 6 strains.

A schematic of the *in vitro* PK/PD model is shown in Fig. S1 in the supplemental material. R1 is the diluent compartment, and R2 is the dosing compartment. The simulated intravenous drug (fosfomycin) was added to the R2 compartment, and the drug that simulated a steady-state concentration (linezolid) was added to R1, R2, and the central compartment to ensure that the concentration of linezolid is stable before the start of the experiment. The whole model was a closed system, during which the volume of the central compartment was constant at 200 mL, and the initial inoculation amount of bacteria was ∼1 × 10^6^ CFU/mL. A peristaltic pump was used to drive the drug-containing or blank medium into the central compartment, and the software WinLIN 3.2 was used to adjust the flow rate of the peristaltic pump in stages to achieve the goal of simulating the drug concentration in human plasma. Owing to the pressure balance, the volume of the outflow liquid was the same as that of the inflow. The bottom of the central compartment was sealed with a 0.45-μm filter membrane to stop the bacteria from flowing out and turn on the magnetic stirrer such that the bacteria and broth in the model were fully mixed. Samples were collected from the central compartment with a syringe at 0, 2, 4, 6, 8, 10, 12, and 24 h, and 10 μL of appropriately diluted sample was manually plated onto MHA for viable cell counting. Enumeration was performed manually after 24 h of incubation at 37°C. The limit of detection (LOD) was set at 100 CFU/mL. Samples were stored in a −80°C refrigerator until drug concentration testing.

Fosfomycin concentrations in the PK/PD model were determined using a biological assay that utilized Escherichia coli ATCC 25922 as an indicator organism (46). Overnight cultures of ATCC 25922 were diluted in MHA supplemented with G-6-P (25 mg/L) to achieve 10^6^ CFU/mL and incubated at 37°C for 18 h. The test samples and quality control samples were tested three times. The calibration curve of standard fosfomycin showed good linearity in the specified concentration range (10 to 400 mg/L) with a correlation coefficient (*R*^2^) greater than 0.99.

### Semimechanical PK/PD model.

The semimechanical PK/PD mathematical model was slightly modified after drawing on a previous method (13–16). A schematic diagram of the PK/PD model of linezolid and fosfomycin is shown in Fig. S2 in the supplemental material. The equations involved in the model are as follows:

(1) $$\frac{dB}{dt}=K_{g}\cdot \left(1-\frac{B}{B_{\max}}\right)\cdot B$$

(2) $$\frac{dB}{dt}=\left[K_{g}\cdot \left(1-\frac{B}{B_{\max}}\right)-E\right]\cdot B$$

(3) $$E=\frac{E_{\max}\cdot C_{\text{drug}}{}^{\text{Hill}}}{{\left(\alpha \cdot {\text{EC}}_{50}\right)}^{\text{Hill}}\;+\;C_{\text{drug}}{}^{\text{Hill}}}$$

(4) $$\text{α}=1+f\cdot \left(1-e^{-\mathrm{ckt}}\right)$$

(5) $$E=E_{\text{LZD}}\cdot {\left[1+\frac{E_{\text{LZD}}}{E_{\text{LZD}}\;+\;E_{\text{FOF}}}\right]}^{\mathrm{int}}+E_{\text{FOF}}\cdot {\left[1+\frac{E_{\text{FOF}}}{E_{\text{LZD}}\;+\;E_{\text{FOF}}}\right]}^{\mathrm{int}}$$

Equation 1 is the growth equation of bacteria, where *K_g_* represents the growth rate of bacteria and *B*_max_ represents the maximum growth value of bacteria. Equation 2 represents the effect of linezolid and fosfomycin on the growth of bacteria, which indicates the change in bacterial quantity with the change in drug concentration. The effects of linezolid and fosfomycin on bacteria conform to the sigmoid *E*_max_ equation (13–15), *E* is the bactericidal effect; *E*_max_ is the maximum achievable kill rate constant; EC_50_ is the drug concentration required to reach half of the *E*_max_; α is the adaptive resistance factor, which is related to both time and drug concentration; and *f* and *k* represent the maximum adaptive resistance factor and the adaptive resistance rate, respectively, as shown in Equations 3 and 4. Equation 5 is the combination drug model, where E represents the combined bactericidal effect of linezolid and fosfomycin, and int represents their interaction. Int > 0, synergy; Int < 0, indifference or antagonism (17).

### Model validation and prediction.

The performance of the final model was first evaluated by visual inspection of the diagnostic goodness-of-fit plots. Goodness-of-fit plots included the following scatterplots: OBS versus population prediction (PRED), OBS versus individual prediction (IPRED), conditional weighted residual errors (CWRES) versus PRED, and CWRES versus time (47). The established model was further validated by a visual predictive check (VPC) (48), which is commonly used to determine whether a model can reproduce the variability and main trend of the observed data. Typically, 1,000 data sets were modeled using Monte Carlo simulations based on the final model parameters. The observed data were then compared with the 2.5th, 50th, and 97.5th percentiles of the simulated data to assess the predictive capacity of the final model. In this study, VPC was stratified by bacterial strains and drugs.

Additionally, the predictive ability of the semimechanical PK/PD model must be externally validated, that is, whether the model developed on known experimental data can predict the pharmacodynamics of new dosage regimens. The computational model was employed to predict the bacterial counts under the dosage regimen of 12 mg/L linezolid and 8 g fosfomycin every 8 h with a 0.5-h infusion. The prediction data were compared with the experimental data, where a combination of linezolid and fosfomycin was administered to ATCC 29212. After successful model validation, simulations were performed using NONMEM to predict the dosing regimen. Linezolid was simulated at the steady-state concentrations (4 to 20 mg/L) achieved in humans at the administered dose of 600 mg q12h, and the concentration (>20 mg/L) outside the safe range after increasing the administered dose was also simulated. Linezolid concentration ranged from 4 to 24 mg/L. Fosfomycin was simulated in the PK section in a two-compartment model with doses of 2, 4, 6, 8, 10, 12, and 14 g every 8 h with a 0.5-h infusion. Predictions were made using mono- and combination therapies.

### Software.

The data were analyzed using the first-order conditional estimation with interaction (FOCE-I) method and ADVAN6 within the population analysis software NONMEM 7.4.1 (level 1.0; ICON Development Solutions, New York, NY, USA). NONMEM was also used to predict the concentration versus time and bacterial count versus time profiles. Plotting was performed using R software (version 3.6.0; The R Foundation of Statistical Computing, Vienna, Austria) and Origin 9.0.

## References

[1] Ch'ng JH, Chong KKL, Lam LN, Wong JJ, Kline KA. 2019. Biofilm-associated infection by *enterococci*. Nat Rev Microbiol 17:82–94. doi:10.1038/s41579-018-0107-z. 30337708 PMC13729018

[2] Chirouze C, Athan E, Alla F, Chu VH, Ralph Corey G, Selton-Suty C, Erpelding ML, Miro JM, Olaison L, Hoen B, International Collaboration on Endocarditis Study Group. 2013. *Enterococcal* endocarditis in the beginning of the 21st century: analysis from the International Collaboration on Endocarditis-Prospective Cohort Study. Clin Microbiol Infect 19:1140–1147. doi:10.1111/1469-0691.12166. 23517406

[3] Shaw KJ, Rather PN, Hare RS, Miller GH. 1993. Molecular genetics of aminoglycoside resistance genes and familial relationships of the aminoglycoside-modifying enzymes. Microbiol Rev 57:138–163. doi:10.1128/mr.57.1.138-163.1993. 8385262 PMC372903

[4] Boak LM, Rayner CR, Grayson ML, Paterson DL, Spelman D, Khumra S, Capitano B, Forrest A, Li J, Nation RL, Bulitta JB. 2014. Clinical population pharmacokinetics and toxicodynamics of linezolid. Antimicrob Agents Chemother 58:2334–2343. doi:10.1128/AAC.01885-13. 24514086 PMC4023770

[5] Chen Q, Yin D, Li P, Guo Y, Ming D, Lin Y, Yan X, Zhang Z, Hu F. 2020. First report *Cfr* and *OptrA* co-harboring linezolid-resistant *Enterococcus faecalis* in China. Infect Drug Resist 13:3919–3922. doi:10.2147/IDR.S270701. 33173316 PMC7646505

[6] Pfaller MA, Mendes RE, Streit JM, Hogan PA, Flamm RK. 2017. Five-year summary of *in vitro* activity and resistance mechanisms of linezolid against clinically important Gram-positive cocci in the United States from the LEADER surveillance program (2011 to 2015). Antimicrob Agents Chemother 61:e00609-17. doi:10.1128/AAC.00609-17. 28483950 PMC5487612

[7] Qi C, Xu S, Wu M, Zhu S, Liu Y, Huang H, Zhang G, Li J, Huang X. 2019. Pharmacodynamics of linezolid-plus-fosfomycin against vancomycin-susceptible and -resistant enterococci *in vitro* and *in vivo* of a *Galleria mellonella* larval infection model. Infect Drug Resist 12:3497–3505. doi:10.2147/IDR.S219117. 31814738 PMC6858807

[8] Jiang L, Xie N, Chen M, Liu Y, Wang S, Mao J, Li J, Huang X. 2021. Synergistic combination of linezolid and fosfomycin closing each other's mutant selection window to prevent enterococcal resistance. Front Microbiol 11:605962. doi:10.3389/fmicb.2020.605962. 33633692 PMC7899970

[9] Boak LM, Li J, Rayner CR, Nation RL. 2007. Pharmacokinetic/pharmacodynamic factors influencing emergence of resistance to linezolid in an *in vitro* model. Antimicrob Agents Chemother 51:1287–1292. doi:10.1128/AAC.01194-06. 17242144 PMC1855482

[10] Yang M, Zhang J, Chen Y, Liang X, Guo Y, Yu J, Zhu D, Zhang Y. 2017. Optimization of linezolid treatment regimens for Gram-positive bacterial infections based on pharmacokinetic/pharmacodynamic analysis. Future Microbiol 12:39–50. doi:10.2217/fmb-2016-0140. 27922745

[11] Abbott IJ, van Gorp E, van der Meijden A, Wijma RA, Meletiadis J, Roberts JA, Mouton JW, Peleg AY. 2020. Oral fosfomycin treatment for enterococcal urinary tract infections in a dynamic *in vitro* model. Antimicrob Agents Chemother 64:e00342-20. doi:10.1128/AAC.00342-20. 32253214 PMC7269476

[12] Aranzana-Climent V, Buyck JM, Smani Y, Pachón-Diaz J, Marchand S, Couet W, Grégoire N. 2020. Semi-mechanistic PK/PD modelling of combined polymyxin B and minocycline against a polymyxin-resistant strain of *Acinetobacter baumannii*. Clin Microbiol Infect 26:1254.e9–1254.e15. doi:10.1016/j.cmi.2020.01.017. 32006693

[13] Schmidt S, Sabarinath SN, Barbour A, Abbanat D, Manitpisitkul P, Sha S, Derendorf H. 2009. Pharmacokinetic-pharmacodynamic modeling of the *in vitro* activities of oxazolidinone antimicrobial agents against methicillin-resistant *Staphylococcus aureus*. Antimicrob Agents Chemother 53:5039–5045. doi:10.1128/AAC.00633-09. 19786607 PMC2786350

[14] Scheerans C, Wicha SG, Michael J, Derendorf H, Kloft C. 2015. Concentration-response studies and modelling of the pharmacodynamics of linezolid: *Staphylococcus aureus* versus *Enterococcus faecium*. Int J Antimicrob Agents 45:54–60. doi:10.1016/j.ijantimicag.2014.07.028. 25455852

[15] Docobo-Pérez F, Drusano GL, Johnson A, Goodwin J, Whalley S, Ramos-Martín V, Ballestero-Tellez M, Rodriguez-Martinez JM, Conejo MC, van Guilder M, Rodríguez-Baño J, Pascual A, Hope WW. 2015. Pharmacodynamics of fosfomycin: insights into clinical use for antimicrobial resistance. Antimicrob Agents Chemother 59:5602–5610. doi:10.1128/AAC.00752-15. 26124169 PMC4538498

[16] Bian X, Liu X, Chen Y, Chen D, Li J, Zhang J. 2019. Dose optimization of colistin combinations against carbapenem-resistant *Acinetobacter baumannii* from patients with hospital-acquired pneumonia in China by using an *in vitro* pharmacokinetic/pharmacodynamic model. Antimicrob Agents Chemother 63:e01989-18. doi:10.1128/AAC.01989-18. 30745385 PMC6437507

[17] Mohamed AF, Kristoffersson AN, Karvanen M, Nielsen EI, Cars O, Friberg LE. 2016. Dynamic interaction of colistin and meropenem on a WT and a resistant strain of *Pseudomonas aeruginosa* as quantified in a PK/PD model. J Antimicrob Chemother 71:1279–1290. doi:10.1093/jac/dkv488. 26850719

[18] Mohamed AF, Cars O, Friberg LE. 2014. A pharmacokinetic/pharmacodynamic model developed for the effect of colistin on *pseudomonas aeruginosa in vitro* with evaluation of population pharmacokinetic variability on simulated bacterial killing. J Antimicrob Chemother 69:1350–1361. doi:10.1093/jac/dkt520. 24474432

[19] Davis H, Brown R, Ashcraft D, Pankey G. 2020. *In vitro* synergy with fosfomycin plus doxycyclin against linezolid and vancomycin-resistant *Enterococcus faecium*. J Glob Antimicrob Resist 22:78–83. doi:10.1016/j.jgar.2020.01.014. 32007618

[20] Chen H, Du Y, Xia Q, Li Y, Song S, Huang X. 2020. Role of linezolid combination therapy for serious infections: review of the current evidence. Eur J Clin Microbiol Infect Dis 39:1043–1052. doi:10.1007/s10096-019-03801-x. 31898798

[21] Yu W, Zhang J, Tong J, Zhang L, Zhan Y, Huang Y, Qiu Y. 2020. *In vitro* antimicrobial activity of fosfomycin, vancomycin and daptomycin alone, and in combination, against linezolid-resistant *Enterococcus faecalis*. Infect Dis Ther 9:927–934. doi:10.1007/s40121-020-00342-1. 32964392 PMC7680468

[22] Hua R, Xia Y, Wu W, Yan J, Yang M. 2018. Whole transcriptome analysis reveals potential novel mechanisms of low-level linezolid resistance in *Enterococcus faecalis*. Gene 647:143–149. doi:10.1016/j.gene.2018.01.008. 29325735

[23] Cojutti P, Pai MP, Pea F. 2018. Population pharmacokinetics and dosing considerations for the use of linezolid in overweight and obese adult patients. Clin Pharmacokinet 57:989–1000. doi:10.1007/s40262-017-0606-5. 29080937

[24] Okazaki F, Tsuji Y, Seto Y, Ogami C, Yamamoto Y, To H. 2019. Effects of a rifampicin pre-treatment on linezolid pharmacokinetics. PLoS One 14:e0214037. doi:10.1371/journal.pone.0214037. 31518346 PMC6743782

[25] Kane Z, Gastine S, Obiero C, Williams P, Murunga S, Thitiri J, Ellis S, Correia E, Nyaoke B, Kipper K, van den Anker J, Sharland M, Berkley JA, Standing JF. 2021. IV and oral fosfomycin pharmacokinetics in neonates with suspected clinical sepsis. J Antimicrob Chemother 76:1855–1864. doi:10.1093/jac/dkab083. 33855449 PMC8212774

[26] Rodríguez-Noriega E, Hernández-Morfin N, Garza-Gonzalez E, Bocanegra-Ibarias P, Flores-Treviño S, Esparza-Ahumada S, González-Díaz E, Pérez-Gómez HR, Mendoza-Mujica C, León-Garnica G, Morfín-Otero R. 2020. Risk factors and outcome associated with the acquisition of linezolid-resistant *Enterococcus faecalis*. J Glob Antimicrob Resist 21:405–409. doi:10.1016/j.jgar.2020.01.010. 32004724

[27] Wardenburg KE, Potter RF, D'Souza AW, Hussain T, Wallace MA, Andleeb S, Burnham CD, Dantas G. 2019. Phenotypic and genotypic characterization of linezolid-resistant *Enterococcus faecium* from the USA and Pakistan. J Antimicrob Chemother 74:3445–3452. doi:10.1093/jac/dkz367. 31504566 PMC6857194

[28] Diep JK, Sharma R, Ellis-Grosse EJ, Abboud CS, Rao GG. 2018. Evaluation of activity and emergence of resistance of polymyxin B and ZTI-01 (fosfomycin for injection) against KPC-producing *Klebsiella pneumoniae*. Antimicrob Agents Chemother 62:e01815-17. doi:10.1128/AAC.01815-17. 29203494 PMC5786778

[29] Zhang X, Bi W, Chen L, Zhang Y, Fang R, Cao J, Zhou T. 2020. Molecular mechanisms and epidemiology of fosfomycin resistance in *enterococci* isolated from patients at a teaching hospital in China, 2013–2016. J Glob Antimicrob Resist 20:191–196. doi:10.1016/j.jgar.2019.08.006. 31422238

[30] Begg EJ, Peddie BA, Chambers ST, Boswell DR. 1992. Comparison of gentamicin dosing regimens using an *in-vitro* model. J Antimicrob Chemother 29:427–433. doi:10.1093/jac/29.4.427. 1607331

[31] Sun X, Vilar S, Tatonetti NP. 2013. High-throughput methods for combinatorial drug discovery. Sci Transl Med 5:205rv1. doi:10.1126/scitranslmed.3006667. 24089409

[32] Wiskirchen DE, Shepard A, Kuti JL, Nicolau DP. 2011. Determination of tissue penetration and pharmacokinetics of linezolid in patients with diabetic foot infections using *in vivo* microdialysis. Antimicrob Agents Chemother 55:4170–4175. doi:10.1128/AAC.00445-11. 21709078 PMC3165366

[33] Stalker DJ, Jungbluth GL. 2003. Clinical pharmacokinetics of linezolid, a novel oxazolidinone antibacterial. Clin Pharmacokinet 42:1129–1140. doi:10.2165/00003088-200342130-00004. 14531724

[34] Bi R, Qin T, Fan W, Ma P, Gu B. 2018. The emerging problem of linezolid-resistant *enterococci*. J Glob Antimicrob Resist 13:11–19. doi:10.1016/j.jgar.2017.10.018. 29101082

[35] Abdelhady W, Mishra NN. 2019. Comparative efficacies of linezolid vs. tedizolid in an experimental murine model of vancomycin-resistant enterococcal (VRE) bacteremia. Front Med (Lausanne) 20:6–31. doi:10.3389/fmed.2019.00031. PMC6391330 30842947

[36] Jacqueline C, Caillon J, Le Mabecque V, Miègeville AF, Ge Y, Biek D, Batard E, Potel G. 2009. In vivo activity of a novel anti-methicillin-resistant *Staphylococcus aureus* cephalosporin, ceftaroline, against vancomycin-susceptible and -resistant *Enterococcus faecalis* strains in a rabbit endocarditis model: a comparative study with linezolid and vancomycin. Antimicrob Agents Chemother 53:5300–5302. doi:10.1128/AAC.00984-09. 19752276 PMC2786336

[37] Moreillon P, Wilson WR, Leclercq R, Entenza JM. 2007. Single-dose oral amoxicillin or linezolid for prophylaxis of experimental endocarditis due to vancomycin-susceptible and vancomycin-resistant *Enterococcus faecalis*. Antimicrob Agents Chemother 51:1661–1665. doi:10.1128/AAC.00744-06. 17353251 PMC1855552

[38] MacGowan AP. 2003. Pharmacokinetic and pharmacodynamic profile of linezolid in healthy volunteers and patients with Gram-positive infections. J Antimicrob Chemother 51:17–25. doi:10.1093/jac/dkg248. 12730139

[39] Rodríguez-Gascón A, Canut-Blasco A. 2019. Deciphering pharmacokinetics and pharmacodynamics of fosfomycin. Rev Esp Quimioter 32:19–24. 31131588 PMC6555163

[40] Bilal H, Peleg AY, McIntosh MP, Styles IK, Hirsch EB, Landersdorfer CB, Bergen PJ. 2018. Elucidation of the pharmacokinetic/pharmacodynamic determinants of fosfomycin activity against *Pseudomonas aeruginosa* using a dynamic *in vitro* model. J Antimicrob Chemother 73:1570–1578. doi:10.1093/jac/dky045. 29506207

[41] Clinical and Laboratory Standards Institute. 2020. Performance standards for antimicrobial susceptibility testing; 30th informational supplement. CLSI M100-S30. Clinical and Laboratory Standards Institute, Wayne, PA.

[42] Chin JN, Jones RN, Sader HS, Savage PB, Rybak MJ. 2008. Potential synergy activity of the novel ceragenin, CSA-13, against clinical isolates of *Pseudomonas aeruginosa*, including multidrug-resistant *P aeruginosa*. J Antimicrob Chemother 61:365–370. doi:10.1093/jac/dkm457. 18079128

[43] Liu X, Zhao M, Chen Y, Bian X, Li Y, Shi J, Zhang J. 2016. Synergistic killing by meropenem and colistin combination of carbapenem-resistant *Acinetobacter baumannii* isolates from Chinese patients in an *in vitro* pharmacokinetic/pharmacodynamic model. Int J Antimicrob Agents 48:559–563. doi:10.1016/j.ijantimicag.2016.07.018. 27670371

[44] Stalker DJ, Jungbluth GL, Hopkins NK, Batts DH. 2003. Pharmacokinetics and tolerance of single- and multiple-dose oral or intravenous linezolid, an oxazolidinone antibiotic, in healthy volunteers. J Antimicrob Chemother 51:1239–1246. doi:10.1093/jac/dkg180. 12668582

[45] Frossard M, Joukhadar C, Erovic BM, Dittrich P, Mrass PE, Van Houte M, Burgmann H, Georgopoulos A, Müller M. 2000. Distribution and antimicrobial activity of fosfomycin in the interstitial fluid of human soft tissues. Antimicrob Agents Chemother 44:2728–2732. doi:10.1128/AAC.44.10.2728-2732.2000. 10991852 PMC90143

[46] VanScoy B, McCauley J, Bhavnani SM, Ellis-Grosse EJ, Ambrose PG. 2016. Relationship between fosfomycin exposure and amplification of *Escherichia coli* subpopulations with reduced susceptibility in a hollow-fiber infection model. Antimicrob Agents Chemother 60:5141–5145. doi:10.1128/AAC.00355-16. 27270274 PMC4997836

[47] Ette EI, Ludden TM. 1995. Population pharmacokinetic modeling: the importance of informative graphics. Pharm Res 12:1845–1855. doi:10.1023/a:1016215116835. 8786955

[48] Post TM, Freijer JI, Ploeger BA, Danhof M. 2008. Extensions to the visual predictive check to facilitate model performance evaluation. J Pharmacokinet Pharmacodyn 35:185–202. doi:10.1007/s10928-007-9081-1. 18197467 PMC2798054

