# Supplementary material for: Dose Optimization of Combined Linezolid and Fosfomycin against Enterococcus by Using an In Vitro Pharmacokinetic/Pharmacodynamic Model
Source: Microbiol Spectr. 2021 Dec 1;9(3):e00871-21. doi: 10.1128/Spectrum.00871-21 (PMC8635129; doi:10.1128/Spectrum.00871-21)
Supplement: SUPPLEMENTAL FILE 1 — Supplemental material. Download SPECTRUM00871-21_Supp_1_seq14.pdf, PDF file, 3.3 MB [file spectrum00871-21_supp_1_seq14.pdf]

TABLE S1 The fosfomycin pharmacokinetic parameters

Parameter	Explanation	Dose(4g,6g,8g)
$K_e(h^{-1})$	elimination rate constant	0.22
$V_1(L)$	volume of central compartment	21.51
$V_2(L)$	volume of peripheral compartment	10.26
$K_{12}(h^{-1})$	rate constant from central compartment to peripheral compartment	0.20
$K_{21}(h^{-1})$	rate constant from peripheral compartment to central compartment	0.41

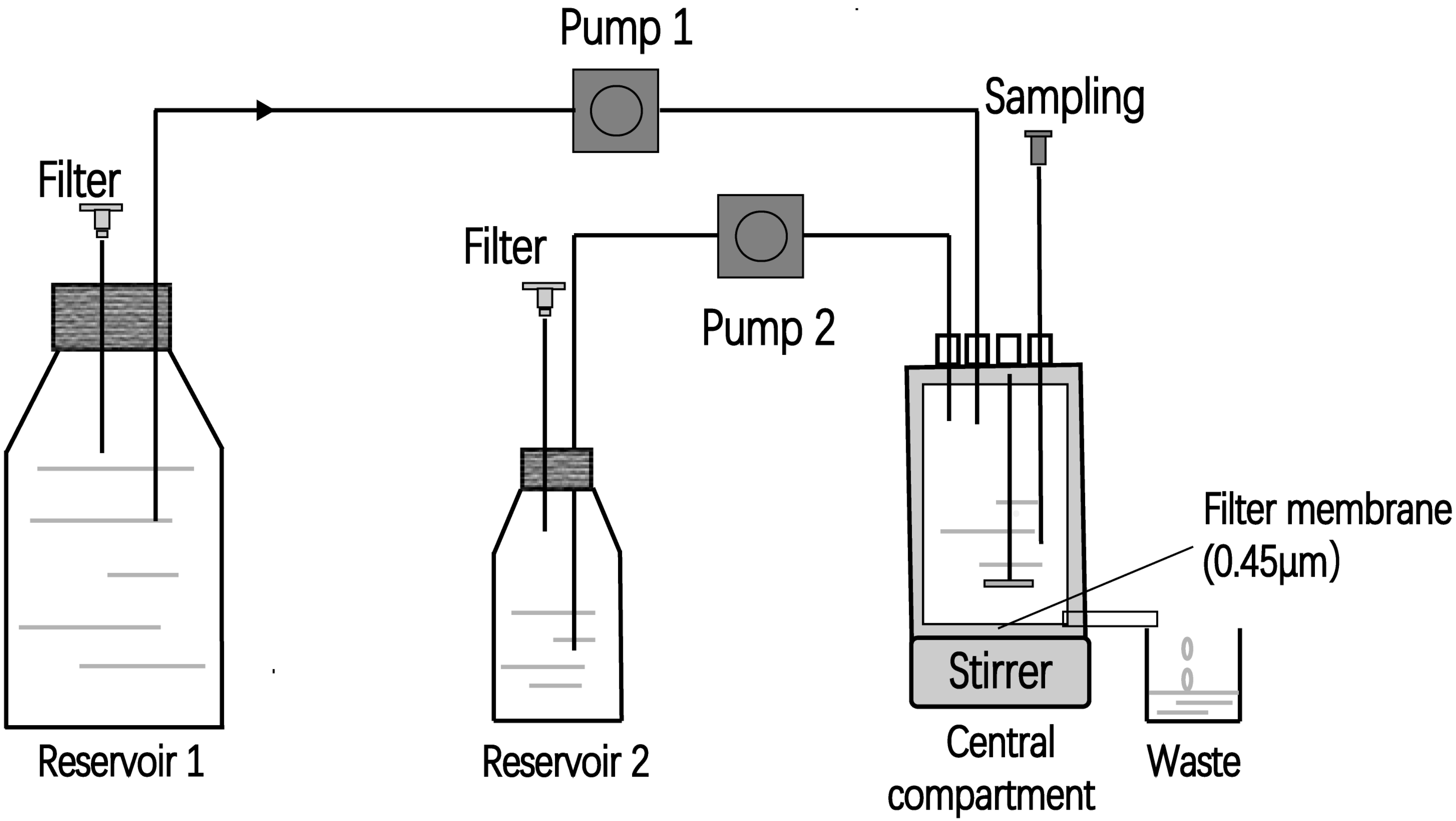
Pump 1
Sampling
Filter
Filter
Pump 2
Filter membrane
(0.45μm)
Stirrer
Reservoir 1
Reservoir 2
Central
compartment
Waste

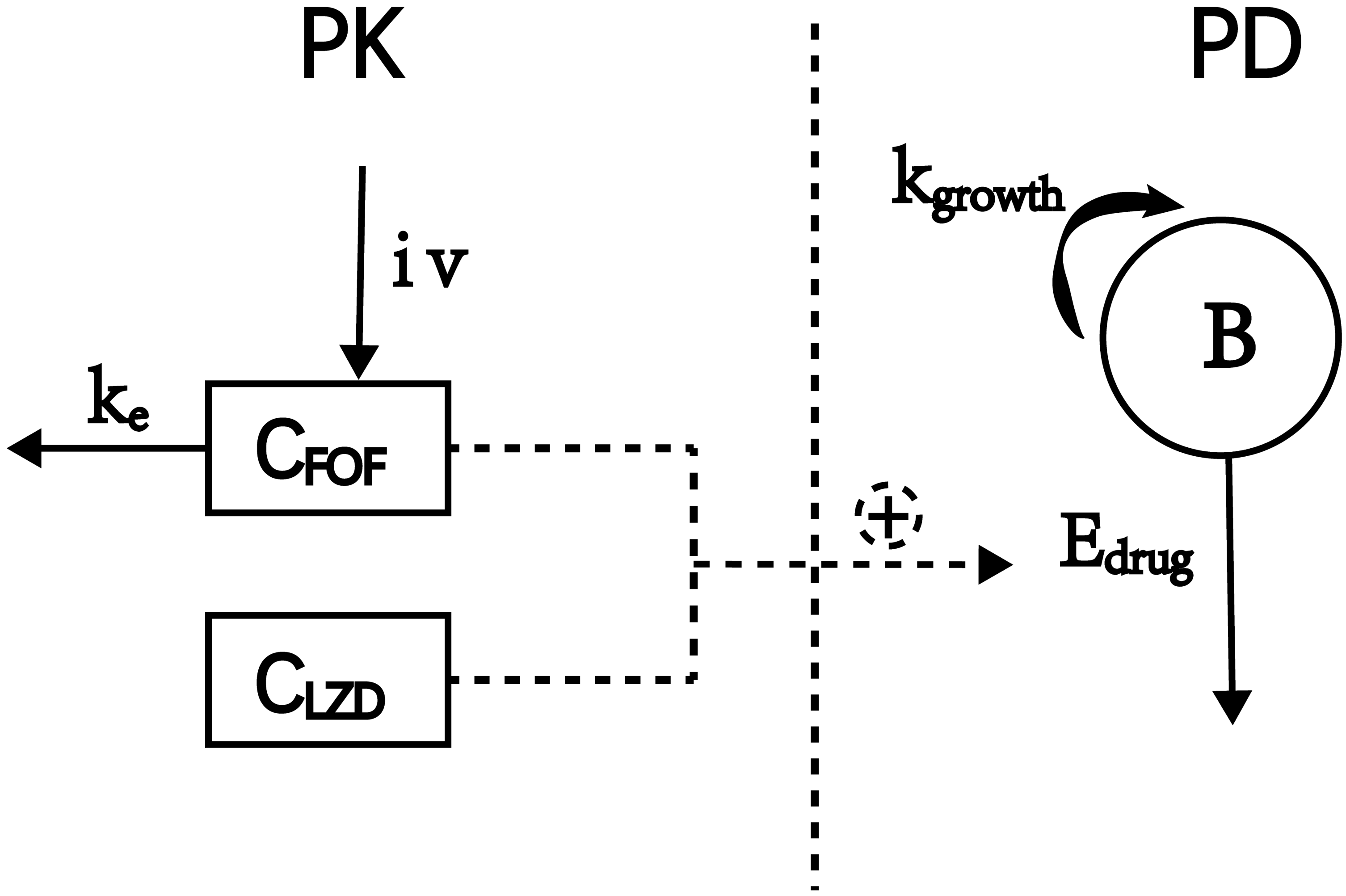
PK
PD
i v
k_e
C_{FOF}
C_{LZD}
k_{growth}
B
+
E_{drug}

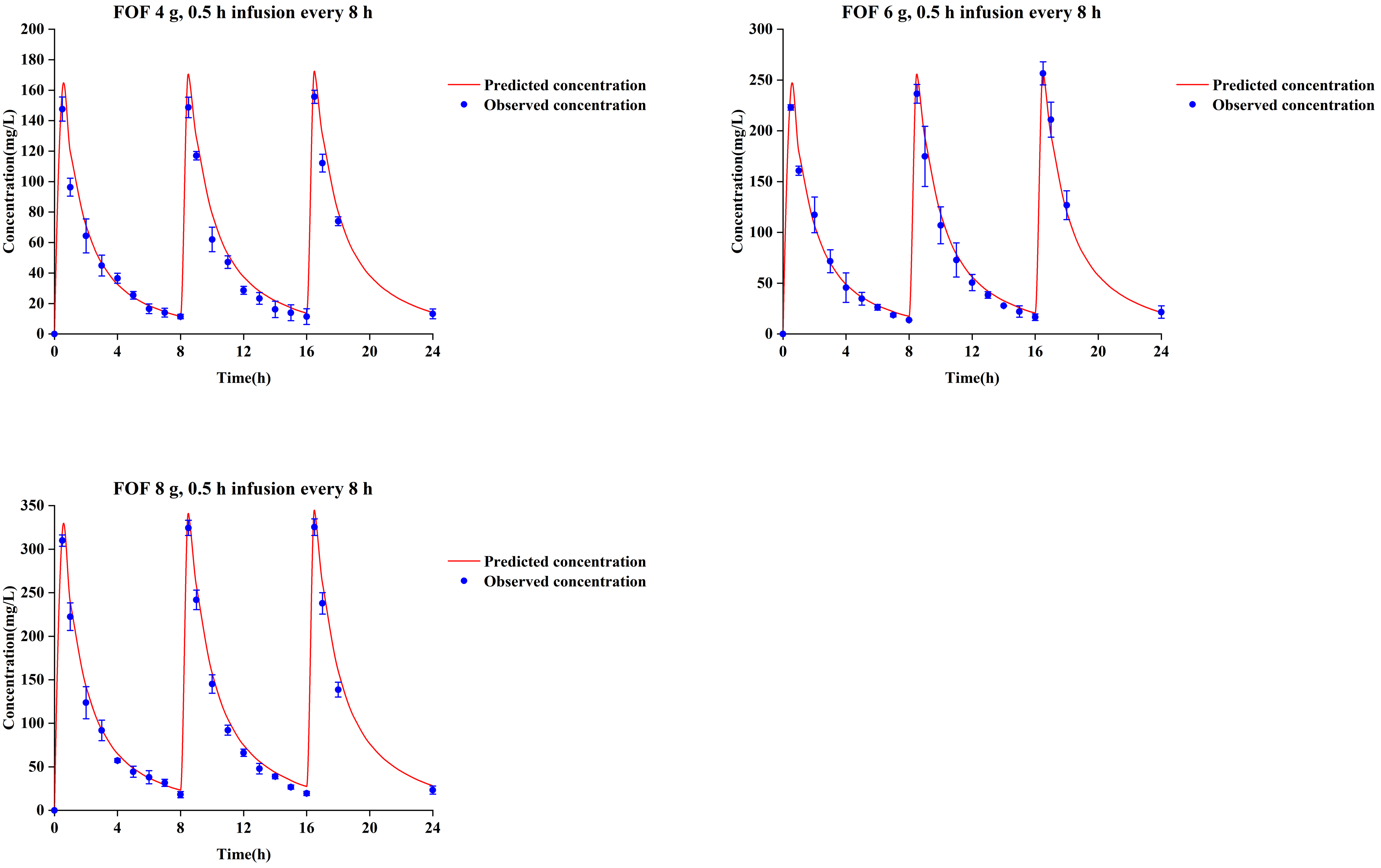
FOF 4 g, 0.5 h infusion every 8 h
FOF 6 g, 0.5 h infusion every 8 h
FOF 8 g, 0.5 h infusion every 8 h
Concentration(mg/L)
Time(h)
Predicted concentration
Observed concentration

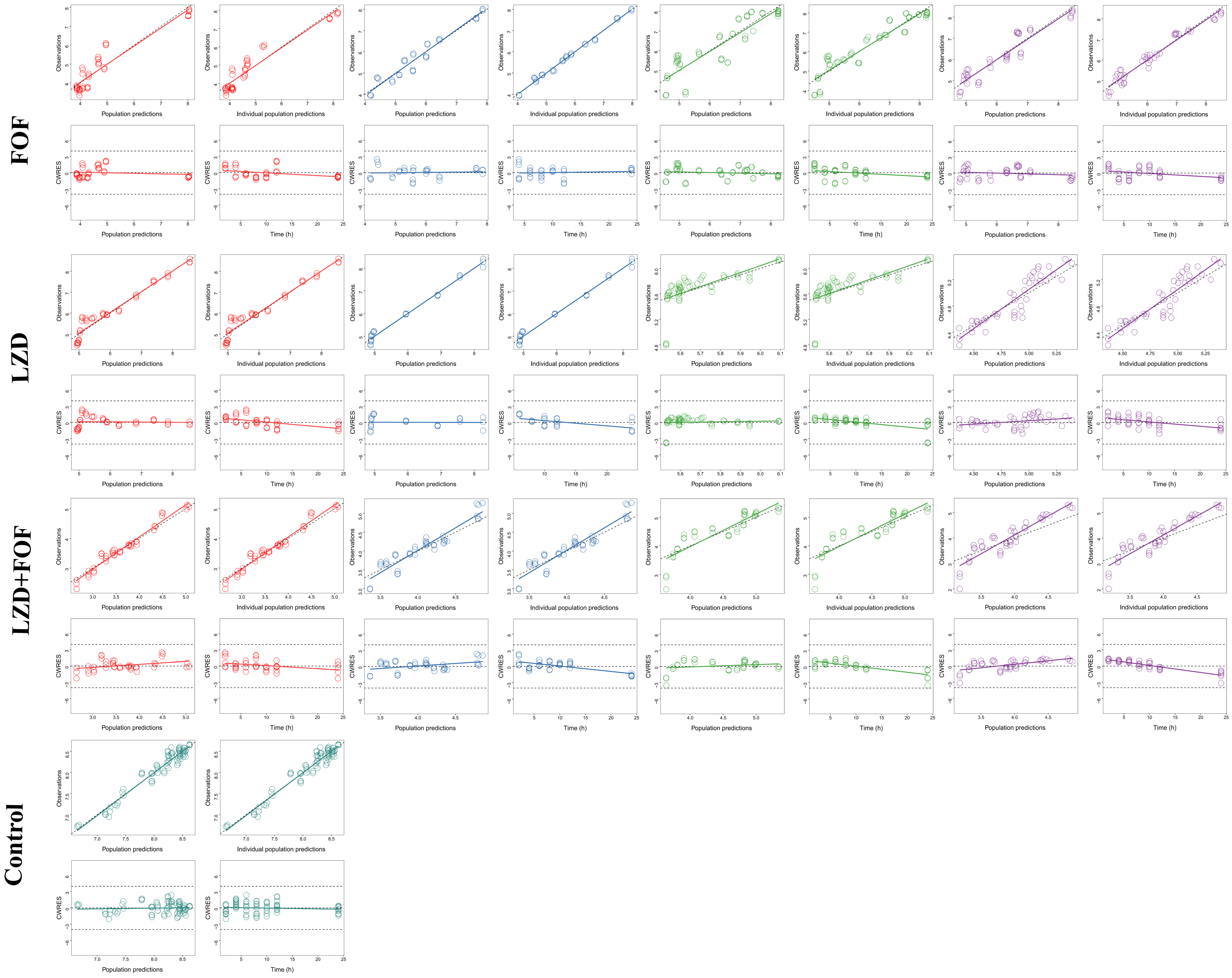
FOF
LZD
LZD+FOF
Control
Observations
CWRES
Population predictions
Individual population predictions
Time (h)

Legends of supplementary materials

TABLE S1 The fosfomycin pharmacokinetic parameters

FIG S1 The schematic of the *in vitro* PK/PD model. R1 is the diluent compartment and R2 is the dosing compartment, the simulated intravenous drug (fosfomycin) was added to the R2 compartment, drug that simulate steady-state concentration (linezolid) was added to R1, R2 and the central compartment to ensure that the concentration of linezolid is constant before the start of the experiment. The peristaltic pump was used to drive the drug-containing or blank medium into the central compartment, and the software WinLIN 3.2 was used to adjust the flow rate of peristaltic pump in every stages to simulate the drug concentration in human plasma. The bottom of the central compartment is sealed with a 0.45 μm filter membrane to prevent the bacteria from flowing out and the magnetic stirrer was used to mix, the bacteria and broth in the model completely.

FIG S2 The mechanism diagram of the semi-mechanical PK/PD model. B stands for bacteria in a self-replicating state, and its net growth rate is expressed by k_g. E_{drug} represents the bactericidal effect of linezolid and fosfomycin on bacteria, C_{LZD} and C_{FOF} represent the real-time concentration of linezolid and fosfomycin, K_e represents the elimination rate of fosfomycin in the body

FIG S3 Comparison of predicted and observed concentrations of fosfomycin *in vitro* PK/PD model. FOF:fosfomycin

Figure S4 Goodness of fit. Each GOF graph contains four small graphs, representing (A) Population predicted value versus observed value. (B) Individual predicted value versus observed value. (C) Conditional weighted residuals versus population predicted value. (D) Conditional weighted residuals versus time. The dashed and solid lines in (A) and (B) represent identity and regression lines, respectively, whereas in (C) and (D), the dashed lines are the position where conditional weighted residual equal 0 and the solid lines are the regression lines. From top to bottom, it represents administration of FOF, LZD, FOF combined with LZD, and control group, respectively. From left to right, the four colors represent bacteria No. 1, No. 2, ATCC 29212 and No. 6, respectively. FOF: Fosfomycin; LZD: Linezolid; FOF+LZD: Fosfomycin combined with Linezolid; Control: no drug.
